# Interaction of connexin43 and protein kinase C-delta during FGF2 signaling

**DOI:** 10.1186/1471-2091-11-14

**Published:** 2010-03-25

**Authors:** Corinne Niger, Carla Hebert, Joseph P Stains

**Affiliations:** 1 Department of Orthopaedics, University of Maryland, School of Medicine, Baltimore, MD, USA

## Abstract

**Background:**

We have recently demonstrated that modulation of the gap junction protein, connexin43, can affect the response of osteoblasts to fibroblast growth factor 2 in a protein kinase C-delta-dependent manner. Others have shown that the C-terminal tail of connexin43 serves as a docking platform for signaling complexes. It is unknown whether protein kinase C-delta can physically interact with connexin43.

**Results:**

In the present study, we investigate by immunofluorescent co-detection and biochemical examination the interaction between Cx43 and protein kinase C-delta. We establish that protein kinase C-delta physically interacts with connexin43 during fibroblast growth factor 2 signaling, and that protein kinase C delta preferentially co-precipitates phosphorylated connexin43. Further, we show by pull down assay that protein kinase C-delta associates with the C-terminal tail of connexin43.

**Conclusions:**

Connexin43 can serve as a direct docking platform for the recruitment of protein kinase C-delta in order to affect fibroblast growth factor 2 signaling in osteoblasts. These data expand the list of signal molecules that assemble on the connexin43 C-terminal tail and provide a critical context to understand how gap junctions modify signal transduction cascades in order to impact cell function.

## Background

Gap junctions are transcellular channels formed by the juxtaposition of two hemichannels, each composed of six connexin monomers, present on adjacent cells. The assembled gap junctions then aggregate to form gap junction plaques. Gap junctional communication maintains metabolic continuity between cells and mediates the rapid and efficient transmission of small molecules, including second messengers among the interconnected cells.

Connexin43 (Cx43) is the principal gap junction protein expressed in osteoblasts and osteocytes, where it has been implicated in transmitting hormonal, mechanical load and growth factor induced signals [1,2]. Mutation of connexins has been implicated in several diseases [3-5]. Point mutations in *GJA1*, the gene encoding the gap junction protein Cx43, result in the human pleiotropic disorder oculodentodigital dysplasia, which includes skeletal manifestations [6]. Mouse models of oculodentodigital dysplasia [7,8] and Cx43 genetic ablation [9-11] have underscored the importance of Cx43 in skeletal function. However, little is known about the molecular mechanisms by which gap junctions regulate the function of skeletal tissues.

Using osteoblast cell lines, we have demonstrated that Cx43 can modulate growth factor responses and signal transduction cascades, leading to altered gene expression and osteoblast function [12-14]. We have recently reported that modulation of Cx43 can affect signal transduction in response to fibroblast growth factor 2 (FGF2) in a protein kinase C-delta (PKCδ)-dependent manner in osteoblasts [14]. PKCδ is a member of the novel PKCs, which, unlike classic PKCs, are calcium-independent but are activated by diacylglycerol and phosphatidylserine. In a previous study, we demonstrated that overexpression of Cx43 in a mouse osteoblast cell line could enhance the transcriptional response of the osteocalcin gene promoter to FGF2 treatment; an effect that could be abrogated by inhibition of PKCδ expression or activity [14]. Conversely, inhibition of Cx43 expression could attenuate the ability of these cells to respond to FGF2. From these data, we hypothesize that Cx43 may recruit PKCδ locally to the gap junction plaque where it can respond to second messengers being propagated through the gap junction channel. Indeed, numerous studies have shown that Cx43 can serve as a docking platform for signal complexes, including β-catenin, src, PKCα and PKCε [15-18]. The novel PKC family member, PKCδ, has not been shown to interact with Cx43. Accordingly, we set out to examine the biochemical interactions between Cx43 and PKCδ as it relates to the effects of FGF2 signaling. By understanding these biochemical interactions, we gain insight into the biologically relevant signals that may be propagated through gap junction channels as well as a greater understanding of how gap junctions regulate signal transduction cascades and, ultimately, cell function.

### Results

#### PKCδ-dependent phosphorylation of Cx43

**Figure 1:**
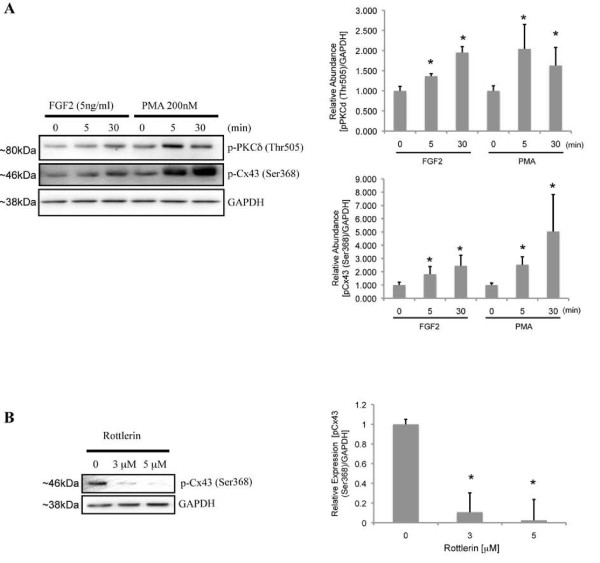
**Phosphorylation of Cx43 at serine 368 by PKCδ**. (A). Western blots (n = 5) performed with anti-phospho-PKCδ (Thr505) and anti-phospho-Cx43 (Ser368) antibodies show concomitant phosphorylation of PKCδ and Cx43 following treatment with 5 ng/ml FGF2 and 200 nM PMA (positive control). (B). Western blots (n = 3) probed with anti-phospho-Cx43 (Ser368) antibodies reveal that the PKCδ inhibitor, rottlerin (3 μM and 5 μM), can block the FGF2-induced phosphorylation of Cx43 at serine 368. Blots probed with anti-GAPDH antibodies are to verify equal loading. Representative blots are shown. Mean densitometric values of the blots from all experiments are shown in the accompanying bar graphs. Error bars indicate standard deviation. An asterisk denotes a p-value <0.05, relative to control.

Whole cell extracts were prepared from MC3T3 osteoblasts following treatment with vehicle (phosphate buffered saline, 0.1% bovine serum albumin, 1 mM dithiothreitol), FGF2 (5 ng/ml) or phorbol 12-myristate 13-acetate (PMA, 200 nM) for up to 30 minutes. Western blots probed with anti-phospho-PKCδ (Thr505) antibodies reveal that treatment with FGF2 (5 ng/ml) increases the phosphorylation of PKCδ by 5 minutes, increasing further by 30 minutes (Figure 1A). Antibodies against phospho-Cx43 (Ser368) reveal that FGF2 treatment induces a concomitant phosphorylation of Cx43 at serine 368, a known target of PKC-mediated phosphorylation (Figure 1A). We observe similar results with PMA treatments, a positive control for PKC phosphorylation at threonine 505 and Cx43 phosphorylation at serine 368 [19]. Pre-treatment of MC3T3 cells with the PKCδ inhibitor rottlerin (3 and 5 μM) prevents the FGF2-induced phosphorylation of Cx43 at serine 368 (Figure 1B). This suggests that PKCδ phosphorylates Cx43 and that the two proteins physically interact, at least transiently, during FGF2 treatment.

### Immunofluorescent detection of PKCδ and Cx43

**Figure 2:**
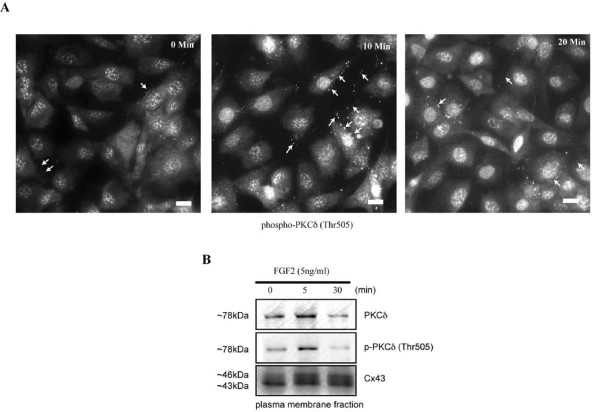
**FGF2 induces the accumulation of phospho-PKCδ in both the nuclei and plasma membrane**. (A). Serum starved MC3T3 cells were subjected to immunofluorescent detection with anti-phospho-PKCδ (Thr505) antibodies during a time course of treatment with FGF2 (5 ng/ml). Representative images collected by epi-fluorescence microscopy are shown for each time point. Small arrows indicate the punctate staining at the cell periphery. The scale bar indicates ~10 μm. (B) Western blots (n = 3) probed with anti-PKCδ, anti-phospho-PKCδ (Thr505) and anti-Cx43 antibodies were performed on equal amounts of plasma membrane protein extracts from FGF2 (5 ng/ml) treated osteoblasts. Representative blots are shown.

In order to examine the subcellular localization of activated PKCδ, MC3T3 cells treated with vehicle or 5 ng/ml FGF2, for the indicated times, were fixed and stained with anti-phospho-PKCδ (Thr505) antibodies (Figure 2A). By immunofluorescence microscopy, we examined the localization of phospho-PKCδ (Thr505) in osteoblasts over a time course following exposure to FGF2. While classic PKCs are rapidly translocated to the plasma membrane upon activation, PKCδ has been shown to translocate not only to the plasma membrane but also to the nucleus [20,21]. Prior to treatment with FGF2 (Figure 2A, 0 min), phospho-PKCδ is detected diffusely throughout the cytoplasm and in defined foci in the nuclei. Occasional, weak staining is observed at the periphery of cells. In contrast, within 10 minutes of exposure to FGF2, there is a rapid accumulation of phospho-PKCδ staining observed in the nuclear compartment (Figure 2A, 10 min). Interestingly, we did not see a generalized staining of the plasma membrane with anti-phospho-PKCδ antibodies, rather we noticed a distinct pattern of staining near the point of cell-cell contacts, that is remindful of the staining we see for Cx43 in these cells. The apparent accumulation of phospho-PKCδ staining at cell-cell contacts remained at 20 minutes following FGF2 stimulation (Figure 2A, 20 min). To determine if PKCδ is accumulating at the plasma membrane, we prepared protein extracts on plasma membrane preparations from osteoblasts during a time course of FGF2 treatment. Subsequent, western blots of these plasma membrane extracts were probed with anti-PKCδ, anti-phospho-PKCδ (Thr505) and anti-Cx43 antibodies. There is a notable accumulation of PKCδ and phospho-PKCδ in the membrane protein extract following treatment with FGF2 for 5 minutes (Figure 2B). This accumulation at the plasma membrane is transient, however, as it begins to diminish 30 minutes after FGF2 administration. Cx43 levels in the membrane fraction remain relatively static (Figure 2B). Importantly, both proteins are found in the plasma membrane fraction, thus allowing for a potential protein-protein interaction.

**Figure 3:**
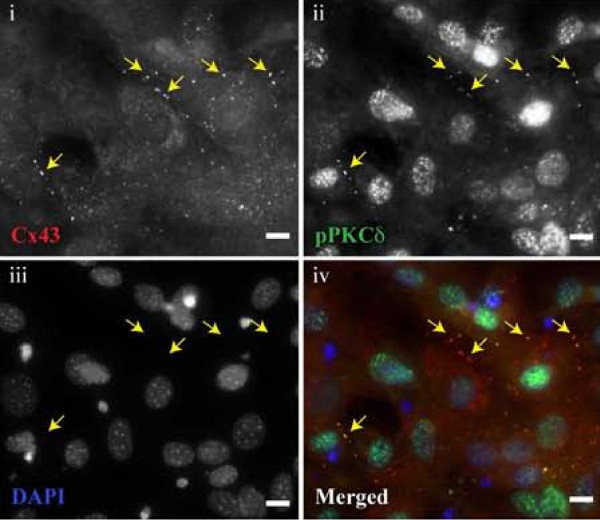
**Co-detection of Cx43 and phospho-PKCδ at the plasma membrane**. Serum starved MC3T3 cells were treated with FGF2 for 10 min prior to fixation and subsequent staining with (i) mouse anti-Cx43 (red), (ii) rabbit anti-phospho-PKCδ (Thr505) (green), (iii) DAPI (blue). A merged image is also shown (iv). Arrows indicate the same position on each panel and correspond to areas where both Cx43 and phospho-PKCδ appear to be closely co-detected in their respective panels. A representative field of view is shown. The white scale bar is ~10 μm.

Given that both proteins are found in the plasma membrane and possess similar patterns of staining, we next sought to see if the pattern of staining observed with phospho-PKCδ antibodies following FGF2 administration was consistent with localization at Cx43 gap junction plaques. Accordingly, we performed immunofluorescent co-detection of both phospho-PKCδ (Thr505) and Cx43. Cx43 is diffusely detected throughout the cytoplasm with distinct areas of bright, punctate fluorescence at the periphery of the cells, near cell-cell borders (Figure 3i). PKCδ is primarily observed at the nuclear compartment of these cells, though a similar pattern of fluorescence accumulation in defined regions at cell-cell borders is observed (Figure 3ii). When the fluorescent images are merged, there is a distinct co-detection of both PKCδ and Cx43 in punctate clusters near cell-cell contacts, consistent with the expected pattern of staining seen at gap junction plaques (Figure 3iv). Importantly, while this immunofluorescent co-detection of PKCδ and Cx43 does not confirm any protein-protein interaction, it provides critical feasibility that the two proteins are located *in situ *in a manner that could permit protein-protein interactions.

### Co-immunoprecipitation of Cx43 and PKCδ

**Figure 4:**
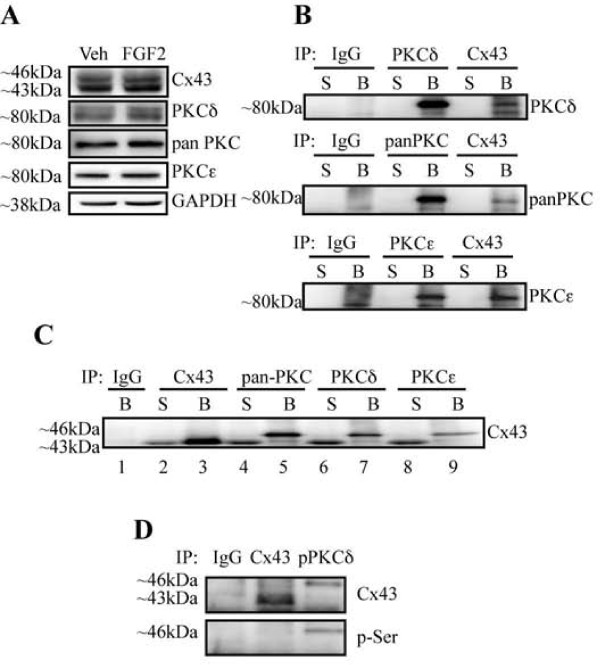
**Connexin43 and PKCδ are present in a protein complex**. (A). Whole cell extracts from cells treated with vehicle or 5 ng/ml FGF2 for 15 minutes were subjected to western blotting with the indicated antibodies (n = 3). (B). Whole cell extracts were immunoprecipitated (IP) with non-immune IgG, anti-PKC (δ, pan or ε) or anti-Cx43 antibodies, as indicated. The supernatant (S) and bead (B) fractions from the immunoprecipitations were subjected to western blotting (WB) with anti-PKC (δ, pan or ε) antibodies. Immunoprecipitation with anti-Cx43 antibodies co-precipitates PKCδ (as well as PKCε). (C). Whole cell extracts were immunoprecipitated with non-immune IgG, anti-Cx43, or anti-PKC (pan, δ, or ε) antibodies, as indicated. The supernatant (S) and bead (B) fractions from the immunoprecipitations were subjected to western blotting with Cx43 antibodies. All three anti-PKC antibodies co-precipitate Cx43. (D). Whole cell extracts were prepared from cells treated with 5 ng/ml FGF2 for 10 minutes. The extracts were immunoprecipitated with non-immune IgG, anti-Cx43, or anti-phospho-PKCδ (Thr505) antibodies, as indicated. The bead fractions from the immunoprecipitations were subjected to western blotting with anti-Cx43 or anti-phospho-serine antibodies. Anti-phospho-PKCδ (Thr505) antibodies preferentially co-immunoprecipitate serine phosphorylated Cx43.

Next, we attempted to establish protein-protein interactions between Cx43 and PKCδ by biochemical means. We performed co-immunoprecipitation experiments from whole cell extracts isolated from MC3T3 cells. Previous studies have shown that PKCα and PKCε can interact with Cx43 [22,23]. As a result, we used an anti-panPKC antibody and an anti-PKCε antibody as controls to ensure the efficacy of our co-immunoprecipitations. First, we confirmed the specificity of the antibodies to be used for co-immunoprecipitation experiments by western blotting MC3T3 whole cell extracts (Figure 4A). As expected, we detect the presence of PKCδ, PKCε and Cx43 in MC3T3 cells. Additionally, the abundance of these factors was not appreciably affected by treatment of the cells for 15 minutes with FGF2 (5 ng/ml). Next, we examined whether anti-Cx43 antibodies could be used to co-immunoprecipitate PKC family members, including PKCδ. Immunoprecipitations were performed with non-immune IgG, anti-PKC or anti-Cx43 antibodies, and western blots of supernatant (S) and bead (B) fractions from the immunoprecipitations were probed with anti-panPKC, anti-PKCδ and anti-PKCε specific antibodies, as indicated in Figure 4B. In all cases, non-immune IgG failed to immunoprecipitate PKCs. As expected, anti-PKC isoform antibodies were able to immunoprecipitate PKCs. Importantly, anti-Cx43 antibodies were able to co-immunoprecipitate proteins detectable with all three anti-PKC antibodies, supporting a protein-protein interaction between PKCs (α, δ and ε) and Cx43.

Next, we attempted the opposing co-immunoprecipitation, using anti-PKC antibodies to co-immunoprecipitate Cx43. Immunoprecipitations were performed with non-immune IgG, anti-Cx43, or anti-PKC antibodies, and western blots of supernatant (S) and bead (B) fractions from the immunoprecipitations were probed with anti-Cx43 specific antibodies, as indicated in Figure 4C. Non-immune IgG failed to immunoprecipitate PKCs. As expected, anti-Cx43 antibodies were able to immunoprecipitate Cx43 protein. Antibodies against panPKC, PKCδ and PKCε precipitate two products that are detected with Cx43 antibodies, a ~43 kD and a ~46 kD protein. The ~43 kD protein is the non-phosphorylated (NP) form of Cx43, while the ~46 kD protein is a phosphorylated form of Cx43 (p-Cx43). Interestingly, it would appear that phosphorylated Cx43 (~46 kD) is preferentially associated with all three PKCs, as this is the principal product detected in the bead fractions from co-immunoprecipitations with anti-PKC antibodies (Figure 4C, lanes 5,7 and 9), in contrast to the predominance of the ~43 kDa band (NP form) that is observed in the Cx43 direct immunoprecipitation (Figure 4C, lane 3).

To validate that this ~46 kD product corresponds to phosphorylated Cx43, co-immunoprecipitations were performed using non-immune IgG, anti-Cx43 and anti-phospho-PKCδ antibodies, and the immunoprecipitated products were probed by western blotting with anti-Cx43 or anti-phospho-serine specific antibodies. The same noticeable enrichment of the higher molecular weight product (~46 kDa) is observed in the co-immunoprecipitations of Cx43 with anti-PKC antibodies in comparison to the direct Cx43 immunoprecipitation. (Figure 4D). In support of the notion that the 46 kDa protein is a phosphorylated form of Cx43, the anti-phospho-serine antibody strongly reacts with the ~46 kDa product in the co-immunoprecipitations with anti-PKCδ antibodies (Figure 4D). These data reinforce the hypothesis that PKCδ preferentially associates with phospho-Cx43.

**Figure 5:**
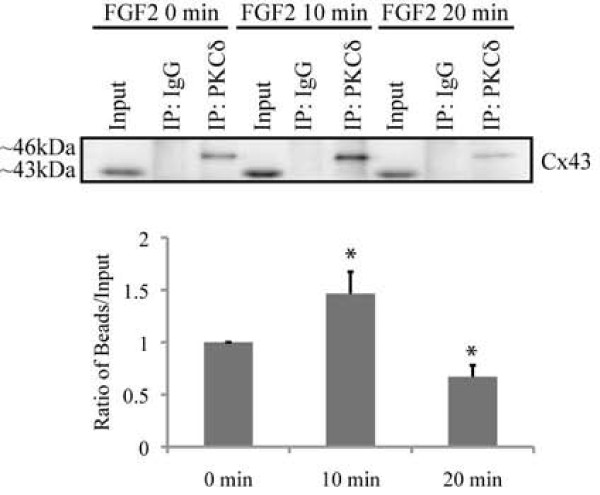
**FGF2 treatment promotes a transient increase in association of Cx43 and phospho-PKCδ**. Whole cell extracts were prepared from cells treated with vehicle or 5 ng/ml FGF2 for the indicated time. The extracts were immunoprecipitated (IP) with non-immune IgG, anti-Cx43, or anti-phospho-PKCδ (Thr505) antibodies, as indicated. A fraction of the input and the bead fractions from the immunoprecipitations were subjected to western blotting (WB) with anti-Cx43 antibodies. The co-precipitated Cx43 and phospho-PKCδ complex is most abundant at 10 minutes and returns to basal levels by 15 minutes. The graph reveals the average ratio of co-immunoprecipitated Cx43 relative to the Cx43 present in the input fraction for each time point from three separate experiments. Error bars indicate standard deviation. An asterisk denotes a p-value <0.05, relative to control. The ratio for the 0 min FGF2 treatment point has been arbitrarily set to a value of 1.

We have previously shown that PKCδ (but not PKCε) translocates to the nucleus of FGF2 treated osteoblasts by 30 minutes [14]. At intermediate time points following FGF2 treatment, there is a distinct accumulation of PKCδ in nodules at the plasma membrane, as shown in Figures 2 and 3. To determine the kinetics of association between Cx43 and PKCδ in FGF2 treated osteoblasts, MC3T3 cells were treated with FGF2 for 0, 10 and 20 minutes. Whole cell extracts were immunoprecipitated with non-immune IgG or anti-PKCδ antibodies and western blots of supernatant (S) and bead (B) fractions from the immunoprecipitations were probed with anti-Cx43 antibodies, as shown in Figure 5. There is a distinct increase in the Cx43/PKCδ interaction following 10 minutes of exposure to FGF2 (~1.5 fold increase in co-immunoprecipitated Cx43 versus input, relative to untreated samples). This association is transient, however, as the Cx43/PKCδ interaction is reduced to below basal levels by 20 minutes (~1.5 fold decrease in co-immunoprecipitated Cx43 versus input, relative to untreated samples). By 40 minutes of FGF2 treatment, we are unable to co-precipitate Cx43 and PKCδ (data not shown). This data is consistent with the kinetics of cellular re-distribution of PKCδ following FGF2 treatment, shown in Figure 2.

### GST pulldown of PKCδ with the Cx43 C-terminal tail

Finally, in order to confirm that the association between PKCδ and Cx43 occurs in the C-terminal domain of Cx43, we performed GST-pulldown assays, using a recombinant GST-Cx43 fusion protein that spans amino acids 241-382 of the Cx43 C-terminal tail (GST-Cx43CT). We anticipated association of PKCδ with the C-terminal tail of Cx43 because this domain is commonly associated with the assembly of signal complexes [15-18] and includes characterized PKC phosphorylation sites [19].

**Figure 6:**
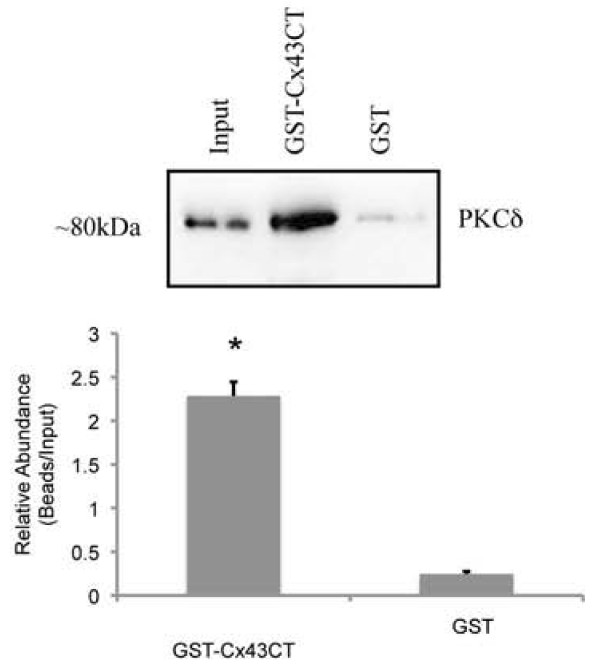
**PKCδ associates with the C-terminal tail of Cx43**. GST pull downs were performed using GST- or GST-Cx43CT(241-381) crosslinked beads and whole cell extracts from MC3T3 cells. The input, and bead fractions were analyzed by western blot. The blots were probed with anti-PKCδ antibodies. PKCδ is pulled down by the GST-Cx43CT(241-381) construct but not by GST-crosslinked beads alone. Mean densitometric values of the blots are shown in the accompanying bar graph. The graph reveals the ratio of "pulled-down" PKCδ relative to the PKCδ present in the input fraction from three separate experiments. Error bars indicate standard deviation. An asterisk denotes a p-value <0.05, relative to control.

Recombinant, purified GST-Cx43CT or GST-alone were crosslinked to glutathione sepharose 4B beads. The crosslinked beads were then used to pulldown interacting proteins from MC3T3 whole cell extracts. The input and bead fractions were then western blotted with anti-PKCδ antibodies. GST-Cx43CT(241-382)-conjugated beads could strongly pull down PKCδ from MC3T3 whole cell extracts (Figure 6). Beads conjugated to GST alone were unable to pull down PKCδ. Specificity of the GST-pulldown was confirmed by probing the western blot with anti-GAPDH antibodies, which revealed an immunoproduct in the input fraction but not in either bead fraction (data not shown).

## Discussion

Previously, we have shown that modulation of Cx43 protein levels can affect the impact of FGF2 on osteoblast transcription in a PKCδ-dependent manner [14]. In that study, we reported that over expression of Cx43 potentiates the transcriptional response to FGF2 by enhancing the activation and mobilization of PKCδ in the cell, which ultimately increases transcription of the osteoblast specific gene, osteocalcin. Conversely, we show that inhibition of Cx43 expression or function could inhibit the responsiveness of the osteocalcin gene to FGF2 treatment. These data established the involvement of Cx43 and PKCδ in the osteoblast response to FGF2, however, it remained undetermined how the relative abundance of Cx43 could impact PKCδ function.

Several studies have shown that FGF2 can activate PKCδ [24-29], or that FGF2 can increase phosphorylation of Cx43 [22,30,31]. Additionally, the activation of PKC isoforms, by phorbol esters has been shown to result in the inhibition of gap junctional communication [19,32-35]. However, a physical interaction of PKCδ with Cx43 has never been reported, nor has FGF2 been shown to increase phosphorylation of Cx43 in a PKCδ-dependent manner.

In this study, we demonstrate via immunofluorescence staining and biochemical methods that PKCδ physically interacts with Cx43. While alone, the immunofluorescence does not prove a physical interaction, we think that this data provides strong support of the feasibility of a physical interaction *in situ *and that the co-precipitation studies are biologically relevant. We go on to characterize the kinetics of the phosphorylation, interaction and cellular translocation following FGF2 treatment, revealing a transient association of PKCδ with Cx43 at the plasma membrane and subsequent accumulation of PKCδ in the nucleus following treatment with FGF2. Our study is the first to show that PKCδ can physically interact with Cx43 in osteoblastic cells, and that there is a PKCδ-dependent phosphorylation of Cx43 at serine 368 that occurs in response to FGF2 administration. It is likely that multiple phosphorylations of Cx43 occur, in addition to the one detected at serine 368, as phosphorylation of serine 368 alone is not sufficient to result in the observed molecular weight shift of Cx43 on SDS-PAGE gels to ~46 kDa [36-38].

A similar interaction between Cx43 and another novel PKC family member, PKCε, has been reported following FGF2 treatment in cardiomyocytes [22]. It is unclear whether the involvement of PKCδ in the FGF2-response and association with Cx43 also occurs in cardiomyocytes, or whether this is an osteoblast-specific interaction. It would not be surprising that PKCδ could also associate with Cx43 in cardiomyocytes, as FGF2, PKCε, PKCδ and Cx43 have all been implicated in cardioprotection following ischemia [39-43]. Notably, in a previously reported study in osteoblasts, we did not detect a translocation of PKCε following FGF2 treatment in these cells, suggesting distinct roles for PKCδ and PKCε in the FGF2-response of bone forming cells [14].

In this study, we report that PKCδ physically interacts with the C-terminal tail of Cx43. We speculate that the preferential interaction of PKCδ with the phosphorylated form of Cx43 is because PKCδ directly phosphorylates Cx43 (i.e., Cx43 is a substrate for PKCδ). The fact that inhibition of PKCδ activity with rottlerin can prevent phosphorylation of Cx43 at serine 368 indicates that, at a minimum, PKCδ lies upstream of Cx43 phosphorylation, and that Cx43 may be a direct substrate for PKCδ. Alternately, it is possible that the phosphorylation of Cx43 is a prerequisite for the docking of PKCδ with the Cx43 C-terminal tail. Future studies will explore these alternatives. It should also be noted that, while commonly used as an inhibitor of PKCδ, the specificity of rottlerin has been questioned [44].

In total, these data support our model that Cx43 affects osteoblast function by regulating signal transduction cascades. We predict that intercellular communication via gap junctions results in the sharing of signals/second messengers among coupled osteoblasts, permitting cells to respond more robustly to extracellular stimuli, such as growth factors, hormonal signals or mechanical load. Central to this model is the concept that Cx43 serves as a docking platform for signaling complexes, recruiting them to the gap junction plaque where they can respond to transmitted signals/second messengers. Previously, we had identified PKCδ as a mediator of the Cx43 enhanced response of osteoblast to FGF2 but, it was unknown whether or not PKCδ was recruited to the Cx43 gap junction plaque to participate in signaling [14]. Now, we can add PKCδ to the growing number of signaling complexes that are recruited to and participate in signaling at the gap junction plaque.

By establishing the protein-protein interactions that can occur between gap junctions and signaling complexes, we gain valuable insights into the molecular mechanisms by which gap junctions/connexins can affect cellular signaling to ultimately affect cell function. Additionally, we learn how gap junctions themselves may be regulated. Indeed, phosphorylation of Cx43 can affect its function/transport, including assembly of hemichannels, transport to the plasma membrane, channel gating and turnover [36,45]. Furthermore, the clarification of these interactions lends insights into the biologically relevant molecules that may be propagated through gap junction channels. The activation of several signaling cascades downstream of the FGF2-FGF receptor complex generates second messengers that may transverse the gap junction channel. Among the signal cascades activated by the FGF2-FGF receptor complex include Ras-ERK MAPK cascade, phospholipase Cγ, phospholipase D and cytosolic phospholipase A2, which produce second messengers like inositol phosphate derivates, diacylglycerol, arachidonic acid, and calcium [46-52]. Of note, several of these second messengers have been shown to regulate PKCδ activation [21,53-56].

Importantly, it is equally plausible that the role of Cx43 in FGF-signaling is independent of its function as a permeable pore for direct cell-cell communications. Connexins have been widely implicated as functioning as unpaired hemichannels [57]. Furthermore, several studies have revealed important non-channel roles for connexins in cell function [58,59]. For example, it is possible that Cx43 may affect FGF2-initiated signaling cascades and osteoblast function by sequestering signal complexes to cellular microdomains, altering mitochondrial function, direct nuclear shuttling or affecting signal complex turnover, independent of cell-cell communication.

Future studies are needed to define the need for PKCδ protein docking with the Cx43 gap junction channel. More specifically, if the proximal recruitment of PKCδ to the Cx43-dependent "shared" messenger is required to transmit the amplification of the FGF2 response among osteoblasts. By studying the mechanism of action of gap junctions in transmitting signals among the cells of bone, we hope to be able to understand the role of gap junctions in how skeletal cells coordinate function during bone growth, remodeling and repair.

## Conclusion

Cx43 can serve as a direct docking platform for the recruitment of PKCδ in order to affect FGF2 signaling in osteoblasts. PKCδ is found to transiently associate with Cx43 at cell-cell junctions, following stimulation with FGF2. Subsequently, PKCδ accumulates in the nucleus, where we have shown in a previous study that it alters transcription of key osteoblastic genes [14]. These data expand the list of signal molecules that assemble on the Cx43 C-terminal tail and provide a critical context to understand how gap junctions modify signal transduction cascades in order to impact cell function.

## Methods

### Chemicals, Antibodies and Reagents

Unless specified, chemicals were purchased from Sigma (St Louis, MO). Recombinant FGF2 was from R&D Systems (Minneapolis, MN). FGF2 was reconstituted in a sterile vehicle solution (phosphate buffered saline, 0.1% bovine serum albumin, 1 mM dithiothreitol). The sources of antibodies used were Sigma (rabbit anti-Cx43 (#C6219) and mouse anti-phospho-serine (#P5747) antibodies), Santa Cruz Biotechnology (Santa Cruz, CA; rabbit anti-panPKC (#sc-10800), rabbit anti-PKCε (#sc-214) and rabbit anti-PKCδ antibodies (#sc-937)), Cell Signaling Technology (Beverly, MA; rabbit anti-phospho-Cx43 (Ser 368) (#3511) and rabbit anti-phospho-PKCδ (Thr505) (#9374), and Chemicon (Temecula, CA; mouse anti-Cx43 (#MAB3067), anti-GAPDH (#MAB374); and Millipore (Billerica, MA) FITC-conjugated donkey anti-rabbit IgG (#AP182F), Cy3-conjugated donkey anti-mouse IgG antibodies(#AP192C). The following reagents were also used: DAPI (4',6-Diamidine-2'-phenylindole dihydrochloride; Roche, Indianapolis, IN), Protein G PLUS/Protein A-Agarose beads (Calbiochem, La Jolla, CA), non-immune mouse and rabbit IgG antibodies (Calbiochem, San Diego, CA).

### Cell Culture and FGF2 Treatments

MC3T3-E1 (clone 4) osteoblasts were purchased from the ATCC (Manassas, VA). These cells were cultured in DMEM (Cellgro, Herndon, VA) supplemented with 10% fetal bovine serum (Hyclone, Logan, UT) and a penicillin (50 IU/ml)-streptomycin (50 μg/ml) solution (Cellgro) as described previously [14]. For experiments with FGF2 treatment, cells were serum-starved in DMEM containing 0.1% fetal bovine serum and 0.3% bovine serum albumin for 24 hours. FGF2 was added to the starvation media at 5 ng/ml. The vehicle diluent for FGF2 (phosphate buffered saline, 0.1% bovine serum albumin, 1 mM dithiothreitol) was used as a negative control for FGF2 treatments. Cell viability was routinely monitored using a CCK-8 assay (Alexis Biochemicals, Farmingdale, NY). Cell viability was unaffected by FGF2 or inhibitor treatments when compared to vehicle treated cells. All experiments performed in this study were repeated at least three times, and data from representative experiments are shown.

### Western Blotting

Whole cell extracts were prepared from confluent cultures of MC3T3 cells using a modified RIPA buffer (50 mM Tris, pH 8.0, 150 mM NaCl, 10 mM sodium pyrophosphate, 10 mM sodium fluoride, 10 mM β-glycerophosphate, 1 mM EGTA, 1 mM EDTA, 2 mM sodium vanadate, 1% NP-40, 0.5% sodium deoxycholate, 0.1% sodium dodecyl sulfate, and 1× protease inhibitor cocktail (Sigma)). For western blots using plasma membrane extracts, plasma membrane fractions were obtained and partially purified using a modified sucrose density gradient method [60]. Briefly, FGF2-treated MC3T3 cells from trypsinized cultures were centrifuged at 900 g at 4°C for 5 minutes and the resulting cell pellets were washed twice in cold HBSS (Cellgro). Cells were resuspended in cold hypotonic buffer (10 mM HEPES, pH 7.9, 1.5 mM MgCl_2_, 10 mM KCl, 0.5 mM DTT) with 1× protease inhibitor cocktail and 1 mM sodium orthovanadate, and incubated on ice for 10 min to allow swelling. Cells were then lysed by adding 1% NP-40. The resulting lysates were centrifuged (1300 g, 10 min., 4°C) to remove unbroken cells and nuclei. The supernatants were then loaded on top of sucrose gradients generated by layering a 10% sucrose (w/v) solution on top of a 60% sucrose solution. Following centrifugation (45,500 g, 30 min., 4°C) enriched plasma membrane fractions were collected in between the 10% and 60% sucrose layers and stored at -80°C until needed. For all western blots, equal amounts of proteins were electrophoresed on 10% SDS-PAGE gels, blotted to PVDF membranes and probed with the indicated antibodies. Membranes were stripped and re-probed with GAPDH antibodies to ensure equal loading of proteins among lanes. All blots were repeated at least three times with independent cell cultures. Representative data are shown for each. Where indicated, the band intensites of western blots were quantitated with ImageJ image analysis software (NIH, Bethesda, MD). Data from multiple blots (a minimum of three) were normalized and averaged. Statistical significance was determined by one-way ANOVA and Tukey's post hoc test. A p-value of <0.05 was used as an indication of statistical significance.

### Immunofluorescence

Cells grown to confluence on coverslips in 6-well plates were prepared for immunostaining, following FGF2 treatments for the indicated time. Samples were stained and imaged as previously described [14]. Staining using non-immune rabbit and mouse IgG and anti-rabbit FITC and anti-mouse Cy3 secondary antibodies were used as a negative control. All immunostaining experiments were performed on a minimum of two coverslips per experiment and repeated on at least three separate cultures. Up to four fields of view were imaged per coverslip. Representative images are shown for each experiment.

### Co-immunoprecipitations

MC3T3 cells were harvested with IP buffer (50 mM Tris pH 8.0, 150 mM NaCl, 1.0% Triton-×-100, 10 mM sodium pyrophosphate, 10 mM α-glycerophosphate, 10 mM sodium fluoride, 1 mM EDTA, 1 mM EGTA, 1 mM sodium orthovanadate and 1× proteases inhibitor cocktail). Whole cell lysates (500 μg total proteins) were pre-cleared with protein A/G agarose beads at 4°C for 60 minutes. The cleared supernatants were incubated overnight at 4°C with 2 μg of the indicated antibodies, followed by a one-hour incubation at 4°C with protein A/G-agarose beads. After five washes in iced cold-PBS, the protein were eluted from the beads by heating the samples for 5 minutes at 95°C in Laemmli SDS buffer (62.5 mM Tris-HCl, 2%w/v SDS, 10% glycerol, 50 mM DTT, 0.01% w/v bromophenol blue). A fraction of the eluted proteins (bead fraction) and supernatant (supe fraction) were then analyzed by western blotting (as described above) with the indicated antibodies.

### Glutathione-S-Transferase (GST) Pulldowns

The region of the GJA1 gene corresponding to the C-terminal tail of the Cx43 protein (amino acids 241-382) were PCR amplified with the following forward and reverse primers: mCx43-EcoR1-241-Forward, GGAATTCCGTCTTCTTCAAGGGCGTT and mCx43-XhoI-382-Reverse, CCGCTCGAGTTAAATCTCCAGGTCAGG. The gel purified Cx43 C-terminal tail PCR product was then subcloned into the *EcoR1*/*XhoI *sites of the pGEX-5X-2 vector (Amersham, Piscataway, NJ) in-frame with an N-terminal GST tag. The resultant pGEX-Cx43CT (241-382) construct was used to generate purified GST-Cx43CT(241-382) protein, as we have published previously [61]. Empty pGEX-5X-2 vector was used to generate GST alone, to be used as a negative control. Equal amounts of purified GST- or GST-Cx43CT(241-382) protein were covalently crosslinked to Glutathione Sepharose 4B beads (Amersham) with dimethyl pimelimidate. Immobilized GST- or GST-Cx43CT (241-382) beads (25 μl) were incubated with 100 μg of MC3T3 whole cell extracts for 4 hours at 4°C. After being washed five times in pulldown buffer (50 mM HEPES, pH 7.5, 1 mM EDTA, 150 mM NaCl, 10% glycerol, 0.1% Tween-20, 0.5 mM DTT, protease inhibitor cocktail), the bound proteins were eluted and analyzed by western blotting.

## Authors' contributions

CN performed the bulk of the experiments JS conceived the idea for this study, constructed the GST-Cx43CT plasmids, and supervised the experiments and data analysis. CH performed the GST-pulldown experiments. JS and CN wrote the manuscript. All authors read and approved the submitted manuscript.
